# Pulsed Laser Deposition of Bismuth Vanadate Thin Films—The Effect of Oxygen Pressure on the Morphology, Composition, and Photoelectrochemical Performance

**DOI:** 10.3390/ma13061360

**Published:** 2020-03-17

**Authors:** Konrad Trzciński, Mariusz Szkoda, Maria Gazda, Jakub Karczewski, Adam Cenian, Galina M. Grigorian, Mirosław Sawczak

**Affiliations:** 1 Faculty of Chemistry, Gdansk University of Technology, Narutowicza 11/12, 80-233 Gdansk, Poland; 2 Faculty of Applied Physics and Mathematics, Gdansk University of Technology, Narutowicza 11/12, 80-233 Gdansk, Poland; 3 Center for Plasma and Laser Engineering, The Szewalski Institute of Fluid-Flow Machinery, Fiszera 14, 80-231 Gdansk, Poland; 4 Physical Department, St. Petersburg State University, 199034 St. Petersburg, Russia

**Keywords:** bismuth vanadate, pulsed laser deposition, photoelectrochemistry

## Abstract

Thin layers of bismuth vanadate were deposited using the pulsed laser deposition technique on commercially available FTO (fluorine-doped tin oxide) substrates. Films were sputtered from a sintered, monoclinic BiVO_4_ pellet, acting as the target, under various oxygen pressures (from 0.1 to 2 mbar), while the laser beam was perpendicular to the target surface and parallel to the FTO substrate. The oxygen pressure strongly affects the morphology and the composition of films observed as a Bi:V ratio gradient along the layer deposited on the substrate. Despite BiVO_4_, two other phases were detected using XRD (X-ray diffraction) and Raman spectroscopy—V_2_O_5_ and Bi_4_V_2_O_11_. The V-rich region of the samples deposited under low and intermediate oxygen pressures was covered by V_2_O_5_ longitudinal structures protruding from BiVO_4_ film. Higher oxygen pressure leads to the formation of Bi_4_V_2_O_11_@BiVO_4_ bulk heterojunction. The presented results suggest that the ablation of the target leads to the plasma formation, where Bi and V containing ions can be spatially separated due to the interactions with oxygen molecules. In order to study the phenomenon more thoroughly, laser-induced breakdown spectroscopy measurements were performed. Then, obtained electrodes were used as photoanodes for photoelectrochemical water splitting. The highest photocurrent was achieved for films deposited under 1 mbar O_2_ pressure and reached 1 mA cm^−2^ at about 0.8 V vs Ag/AgCl (3 M KCl). It was shown that V_2_O_5_ on the top of BiVO_4_ decreases its photoactivity, while the presence of a bulk Bi_4_V_2_O_11_@BiVO_4_ heterojunction is beneficial in water photooxidation.

## 1. Introduction

Bismuth vanadate is one of the most promising candidates for visible light photoelectrochemical water splitting [1]. It is characterized by a moderated energy band gap [2] and high absorption coefficient [3] allowing material to be efficiently excited by visible light. The appropriate flat band potential makes BiVO_4_ a good candidate as a photoanode for efficient water oxidation [4]. It was estimated that BiVO_4_ illuminated by AM1.5 solar light should generate 7.5 mA per cm^2^ [5]. The theoretical efficiency of water photooxidation has not been achieved yet. This is related to the adverse phenomena such as bulk e^−^/h^+^ recombination due to the poor hole mobility and recombination on the surface states. There are many literature reports about BiVO_4_ modifications that lead to the enhancement of its performance [6]. The most common ways consists of Mo and W doping [7] and heterostructures formation [8]. Up to now, the highest photocurrent densities were achieved for bulk (i) W-doped BiVO_4_/V_2_O_5_ heterojunction (6.6 mA cm^−2^) and (ii) electrode material that was prepared on a scaffold of polystyrene spheres scaffold and consisted of WO_3_ and molybdenum doped BiVO_4_ nanoparticles (5.8 mA cm^−2^), both covered by a FeOOH/NiOOH dual layer catalyst [9,10]. However, the theoretical value of the photocurrent should be possible to achieve for “bare” BiVO_4_. Thus, it is still worthwhile to focus on the optimization of BiVO_4_ synthesis and layer preparation. In the literature, one can find different techniques to obtain bismuth vanadate films acting as photoelectrodes, e.g., electrostatic spray pyrolysis [11], deposition of inverse opals using polystyrene spheres as a template [12], simple dip-coating method [13], polymer-assisted spin coating [14], and most promising pulsed laser deposition technique (PLD) [15]. The PLD is a common method of photoactive material deposition [16]. The sputtering conditions during PLD strongly affect the properties of the resulting layer [17]. It was shown that pulsed laser deposited BiVO_4_ films on FTO substrates obtained at 230 °C can generate high photocurrents equal to about 3 mA cm^−2^, but only in the hole-scavenger containing electrolyte [18]. Other authors showed that the high temperature (500 °C) in the deposition chamber makes the growth of BiVO_4_ crystallites anisotropic [19]. The specific orientation and (001) facet exposition facilitates the photoexcited charge carriers transport through the layer and enhance the efficiency of photocurrent generation. A similar effect of BiVO_4_ anisotropic growth was observed for the films deposited at 550 °C on the yttria-stabilized zirconia (YSZ), however the activity of the electrodes in water photooxidation reaction was unsatisfactory [20]. Since the ambient gas pressure strongly affects the expansion dynamic of plasma plume, one may expect different properties of films deposited under different pressures [21]. Indeed, it was reported that the PLD technique can be applied in order to form type-II heterojunction (BiVO_4_/Bi_4_V_2_O_11_) on specifically oriented substrates using stoichiometric BiVO_4_ as a target and the composition of a resulting film can be tuned by changing oxygen pressure [22]. The presence of oxygen in the deposition chamber is crucial to obtain metal oxides films using PLD. The deviations from stoichiometry were observed, e.g., for ZnO [23]. Authors showed that Zn:O ratio was close to one only in the case of the deposition performed in oxygen atmosphere, and the oxygen depletion was observed for process performed in Ar and in vacuum. Oxygen deficiency was also reported for SiO_2_ films deposited in vacuum [24]. Thus, in order to avoid this effect, all depositions of BiVO_4_ layers were carried in oxygen atmosphere. Some authors claimed that the excess of Bi in the target is necessary to get stoichiometric BiVO_4_ films on YSZ substrates [25]. The PLD technique carried out by sequential ablation was also utilized in order to prepare BiVO_4_/WO_3_/SnO_2_ heterojunction, however deposition parameters were not thoroughly investigated [26].

This work is focused on the influence of oxygen pressure in the chamber during pulsed laser deposition on the composition, morphology, and photoelectrochemical properties of bismuth vanadate. Bismuth vanadate layers were deposited on the commercially available FTO substrates from BiVO_4_ pellet (equimolar amounts of bismuth and vanadium). The substrate was placed in the chamber parallel to the laser beam. Such geometry is not chosen in order to obtain the highest possible photoelectrochemical activity of the deposited layer. The proposed geometry allows the spatial distribution of elements deposited on the substrate to be investigated. The results obtained from such geometry of the system give new insight into the mechanism of bismuth vanadate ablation from stoichiometric BiVO_4_ targets. The resulting films were characterized using XRD, Raman spectroscopy, energy dispersive X-ray spectroscopy, and scanning electron microscopy. The photoelectrochemical measurements were performed in an aqueous electrolyte under AM1.5 simulated solar illumination in order to investigate the influence of deposition parameters on the efficiency of photoelectrochemical water oxidation.

## 2. Materials and Methods

### 2.1. Preparation and Characterization of the Target

**Figure 1:**
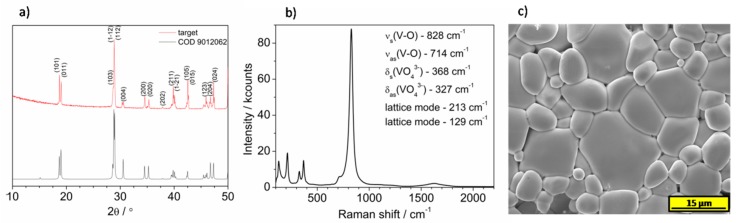
(**a**) The comparison of XRD patterns of target and reference pattern of BiVO_4_ (from crystallography open database 9012062). (**b**) The Raman spectrum of BiVO_4_ target. (**c**) SEM micrograph of annealed BiVO_4_ target.

Bismuth vanadate targets were prepared via conventional solid state reaction using NH_4_VO_4_ (Sigma) and Bi(NO_3_)_3_۰6H_2_O (Sigma) as precursors. Equimolar amounts of Bi and V sources were ground in a mortar and pestle. The mixture was dried overnight at 100 °C and then pressed into a cylindrically shaped pellet with diameter 10 mm using hydraulic press (Specac) with a load of about 64 MPa for about 120 s. The pellet was annealed in static air at 500 °C (tubular furnace, Czylok) in order to transform precursors into BiVO_4_ and get rid of NH_4_NO_3_ formed during the reaction. Then, the material was reground and mixed with poly(ethylene oxide), (Sigma, Mw 300,000) in a mass ratio 50:1 (BiVO_4_:PEO). The polymer (PEO) facilitates the formation of mechanically stable pellets obtained using a hydraulic press (32 MPa, 120 s). These prepared pellets were sintered at 700 °C for 10 h in order to prepare a target for pulsed laser deposition. The high temperature causes decomposition of polymeric binder and the resulting target consists of pure BiVO_4_. The crystal structure of the targets was studied using XRD technique. As is shown in Figure 1a, the proposed method of target preparation leads to the formation of single-phase monoclinic BiVO_4_. Moreover, the Raman spectroscopy technique confirmed that BiVO_4_ pellet is free from carbon residues coming from a binder, see Figure 1b. All bands present in Raman spectrum originate from BiVO_4_ structure. Peaks at Raman shifts below 250 cm^−1^ can be prescribed to lattice modes. Bands at 320–370 cm^−1^ range and 680-915 cm^−1^ range are coming from V–O bending and stretching vibrations, respectively. The morphology of the BiVO_4_ target was examined using SEM. The SEM micrograph of the annealed target is presented in Figure 1c. It can be seen that the pellet is homogenous and contains irregular particles of BiVO_4_ with diameters in a range from about 3 to 22 μm.

### 2.2. Layers Deposition

**Figure 2:**
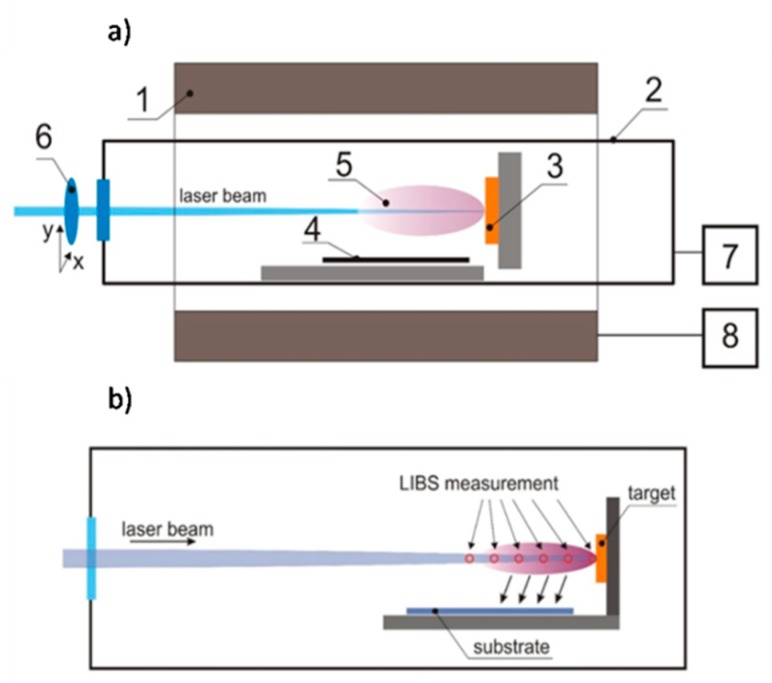
(**a**) Scheme of the pulsed laser deposition technique (PLD) setup: (1) heating chamber, (2) quartz process chamber, (3) target, (4) substrate, (5) laser ablation plume, (6) focusing lens mounted on xy stage, (7) pressure and process gas-composition control-unit, and (8) temperature controller. (**b**) Scheme of the pulsed laser deposition zone with marked positions for laser-induced breakdown spectroscopy (LIBS) measurements.

The bismuth vanadate films were deposited in the specially developed PLD chamber under conditions of controlled temperature and process gas pressure. The scheme of the deposition chamber is shown in Figure 2a. The target (3) and substrate (4) are oriented perpendicularly to each other and mounted in a quartz vacuum chamber (2). The temperature inside the vacuum chamber is controlled with the cylindrical heating system (1) and the temperature control unit (8). The temperature during the deposition (500 °C) is controlled by means of a k-type sensor mounted inside the vacuum chamber. The ablation process is performed using a 266 nm, 6 ns pulse-duration laser-beam of Nd: YAG laser equipped with a FHG module (Brilliant B, Quantel, Newbury, UK). The energy density of the laser was established at about 2.5 J cm^2^. The laser beam is focused on the target surface with the quartz lens (6) mounted on the xy stage to allow scanning of the target surface and avoidance of crater formation. The gas pressure and composition are controlled by means of a vacuum pump and gas dosing valve (7). The pressure of the oxygen inside the deposition chamber was adjusted in the range from 0.1 to 2 mbar depending on the deposition-process conditions. The self-made system for pulsed laser deposition is presented in Figure S1. A part of the samples was prepared in order to measure the gradient of the films thickness (a part of the substrate parallel to the plasma plume was covered). The thickness of the films was estimated using the DektakXT profilometer (Bruker, Billerica, MA, USA). The thickness of the layers did not change significantly with oxygen pressure, see Table S1 in Supporting Information. The results from the profilometer were also confirmed by a cross-sectional SEM micrograph. The exemplary image is presented in Figure S2. It can be concluded, that the thickness of the film roughly equals to the size of single crystallite. Atomic force microscopy (AFM) measurements were performed using Easy Scan 2 microscope (Nanosurf, Liestal, Switzerland) working in the contact mode in order to investigate the topography of deposited films. Films were prepared under different oxygen pressures: 0.1, 0.25, 0.5, 1, and 2 mbar. All samples were deposited at 500 °C for 15 min. Degreased FTO substrates were placed 5 mm from the target parallel to the laser beam. The area of the deposited layers was equal to 0.56 cm^2^ (0.8 cm × 0.7 cm).

### 2.3. Plasma Analysis

In order to study the processes occurring in the laser-induced plasma, especially ionization and recombination processes of ablated species during interaction with oxygen molecules, the LIBS (laser-induced breakdown spectroscopy) measurements were undertaken. The detection of plasma emission was realized using 0.3 m monochromator (SR303i, Andor, Belfast, UK) equipped with 600 grooves/mm grating and intensified charge-coupled device (i-CCD) detector (DH740, Andor). Spectra were recorded in the range of 260–800 nm. Each spectrum was recorded in time resolved mode and averaged from 100 acquisitions. The plasma emission was recorded at different distances from target (0, 5, 15, 15 and 20 mm) as shown in Figure 2b.

### 2.4. Apparatus

Raman spectroscopy measurements were performed using a InVia spectrometer (Renishaw, Wotton-under-Edge, UK) with green laser excitation (514 nm) using a 100× objective and 5% of total laser power. The measurement spot was round-shape with diameter of about 2 μm. XRD patterns were recorded using the diffractometer (Xpert PRO-MPD, Philips, Amsterdam, The Netherlands) with copper Kα radiation (8.04 keV). The morphology of the samples was investigated by Schottky field emission scanning electron microscopy (Quanta FEG 250, FEI, Hillsboro, OR, USA) with an ET (Everhart—Thornley) secondary electron detector. The beam accelerating voltage was kept at 10 kV for films and 20 kV for targets analysis.

## 3. Results and Discussion

**Figure 3:**
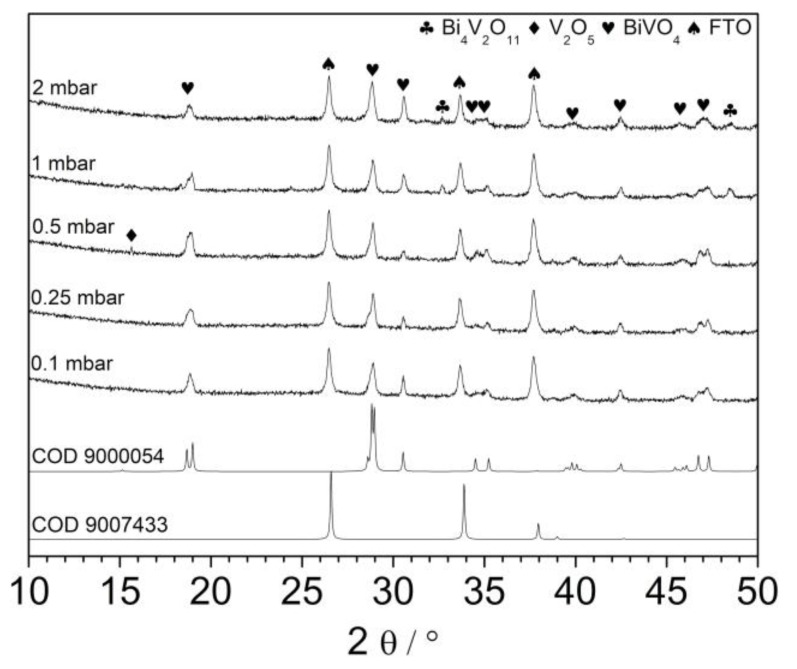
The comparison of XRD patterns recorded for FTO/BiVO_4_ samples deposited at various oxygen pressures. B and V symbols denote Bi_4_V_2_O_11_ and V_2_O_5_, respectively. COD 9000054—BiVO_4_ and COD 9007433—SnO_2_.

The prepared films were characterized using an XRD technique. As it is shown in Figure 3, all patterns exhibits typical reflection characteristic for monoclinic bismuth vanadate. Recently, it was reported that deposition temperature significantly affects the orientation of BiVO_4_ crystallites growing on the FTO substrates [19]. Authors showed that oxygen pressure does not change significantly the crystal structure of resulting layers. Indeed, the presented diffractograms differ rather slightly. Differences may result i.a. from the different thicknesses of the layers and the relative intensity of peaks coming from the FTO substrate. Patterns consist of all the reflexes characteristic for the monoclinic BiVO_4_ phase. However, in the case of the sample deposited under 0.5 mbar, reflex at about 15.5° was detected (V). It could be related to the (002) plane of BiVO_4_ suggesting anisotropic growth of the crystallites, but the intensity of the peak recorded at 30.5° coming from (004) plane was not enhanced. Thus, it is very likely that it is coming from V_2_O_5_ (020) plane [27]. The exemplary XRD pattern recorded in a small-angle scattering mode is shown in Figure S3 for the samples deposited at 0.1, 0.5, and 2 mbar. The patterns are characterized by a much lower intensity of reflections from the FTO substrate as expected. Generally, the same reflexes have been recorded as in the case of conventional XRD analysis. However, the reflection coming from V_2_O_5_ (010) plane at 20.4° was clearly detected for the sample deposited under 0.5 mbar. It confirms that the V_2_O_5_ phase was present rather on the top of the thin film. Interestingly, the peak from a plane (200) (15.5°) was not detected, in contrast to the conventional XRD measurements. It may suggest a very specific arrangement of V_2_O_5_ crystallites. Additionally, the XRD patterns of samples deposited under 1 and 2 mbar consisted of peaks at 32.5° and 48.5° suggesting the coexistence of the Bi_4_V_2_O_11_ (bismuth vanadate with Bi:V ratio 2:1), see Figure 3. Thus, the oxygen pressure in the deposition chamber affects the composition of resulting films. Such a conclusion was already formulated previously. However, the Bi_4_V_2_O_11_ phase crystallized at low O_2_ pressure (unlike in this work), while the application of higher pressure in the deposition chamber led to the formation of pure BiVO_4_ [22]. The differences may result from (i) different substrates and (ii) different geometry of the deposition system. The crystallite size of the films was estimated on the basis of broadening of the XRD reflections. The crystallite size in (101) direction estimated from (101) reflection observed at 18.8° was between 30 and 40 nm. The largest crystallites were observed in the sample obtained at 0.1 mbar, whereas the crystallite size values in the other films were similar (0.25–2 mbar). On the other hand, the reflection at 28.8° corresponding to the (103) and (112) planes was narrower, the crystallite sizes were between 55 and 65 nm. Additionally, in this case, low oxygen partial pressure lead to the formation of larger crystallites.

**Figure 4:**
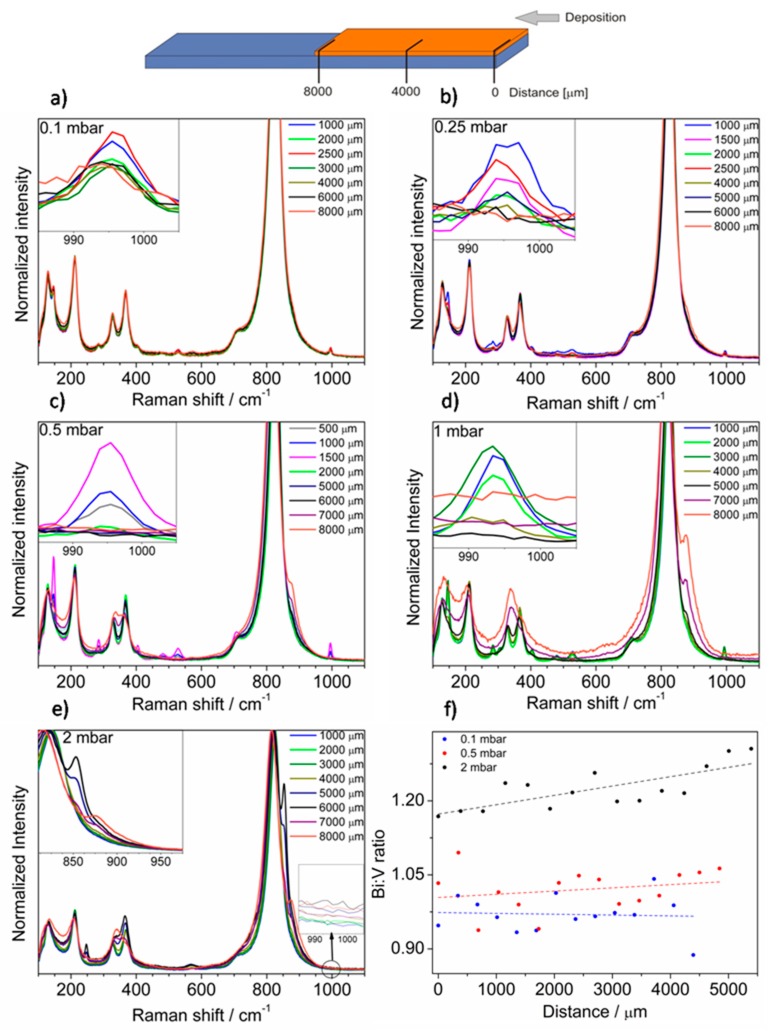
(**a**–**e**) Raman spectra of FTO/BiVO_4_ samples deposited under different pressures measured at different spots and (**f**) Bi:V ratio estimated on the basis of EDX measurements.

Samples have been investigated using the Raman spectroscopy technique. The films showed inhomogeneity and spectra vary depending on the measurement spot distance from the edge of the sample. Thus, the spectra were measured every 500 μm from the edge that was the closest to the target during sputtering. Only in the case of the sample deposited under 0.1 mbar pressure, the film is homogenous, and exemplary spectra are presented in Figure 4a. However, besides bands coming from BiVO_4_, which are characterized by enhanced intensity due to the Raman resonance effect, the new low intensity bands are seen in the Raman spectra at 145, 284, 304, 404, 481, 528, and 995 cm^−1^. All these bands confirm the presence of V_2_O_5_. Interestingly, V_2_O_5_ was not registered on this sample using the XRD measurement, suggesting only short-range ordering in V_2_O_5_ or too small of an amount of it on the sample. Thus, the proposed method of deposition using PLD at certain O_2_ pressure leads to the formation of BiVO_4_/V_2_O_5_ heterojunction on the whole considered surface. The Raman spectra of the film deposited at higher oxygen pressure (0.25 mbar) are presented in Figure 4b. It can be concluded that V_2_O_5_ was not evenly distributed throughout the film. The highest intensity of the band coming from V_2_O_5_ (995 cm^−1^) was detected 1000–2500 μm from the edge. In the further part of the layer, V_2_O_5_ does not occur and only bands originating from BiVO_4_ were detected. Samples obtained at pressure 0.5 mbar exhibited a similar distribution of V_2_O_5_ on the surface, see Figure 4c. The spectra recorded on the spots between 3000 and 6000 μm from the sample edge were characteristic for the pure BiVO_4_ phase. Additionally, the spectrum measured at the opposite site of the layer exhibited a new feature (7000–8000 μm). The bands of BiVO_4_ merged and a new band at about 870 cm^−1^ appeared. In the case of the sample deposited under 1 mbar the effect of peaks merging was enhanced and visible over a larger area of the sample (5000–8000 μm), while maintaining the presence of V_2_O_5_ on the opposite site of the layer (1000–3000 μm), see Figure 4d. Notably, two phenomena do not coexist on any of the same spots of the sample and there is a “buffer layer” containing only BiVO_4_ (3000–5000 μm). In the case of the sample deposited at 2 mbar, the V_2_O_5_ was not detected, see Figure 4e. The spectra collected closest to the edge of the sample (0–4000 μm) consisted of bands characteristic only of BiVO_4_. However, spectra change with distance from the sample edge and new bands at 247 and 567 cm^−1^ appear. Additionally, the two bands characteristic of V–O stretching vibration (800–900 cm^−1^ range) were clearly separated. Thus, depending on the pressure of oxygen in the deposition chamber, the composition of resulting films varied. For the low oxygen pressure, the effect of V_2_O_5_ formation was enhanced. The V_2_O_5_ peaks appeared closer to the sample edge that was closest to the target during deposition. In the case of higher pressures, the V_2_O_5_ disappeared and the opposite side of the sample contains a new phase that can be described as Bi-rich bismuth vanadate—Bi_4_V_2_O_11_. The results obtained suggest that ablation performed using UV laser leads to the generation of plasma plume where Bi and V atoms/ions can be separated. The oxygen pressure may strongly affect V atoms/ions in plasma plume, but heavier Bi atoms/ions were not significantly slowed down due to interactions with oxygen molecules in the deposition chamber. As a result, depending on the oxygen pressure, the V-rich phase (V_2_O_5_) can be formed on the one side of the sample, while the Bi-rich phase (Bi_4_V_2_O_11_) on the opposite. The effect of nonstoichiometric plasma plume propagation due to the differences of cations masses has been already reported for pulsed laser deposition of LaAlO_3_ and LaGaO_3_ [28,29]. It was also reported on the basis on Langmuir probe measurements that lighter Cu_+_ ions are more efficiently slowed down by collisions with oxygen molecules than heavier Ba^+^ and Y^+^ ions during deposition of YBa_2_Cu_3_O_7_ [30]. EDX (energy-dispersive X-ray spectroscopy) scans were performed in order to study the Bi:V atomic ratio along the layer. The results for layers obtained under 0.1, 0.5, and 2 mbar are presented in Figure 4f. The spectra were measured point by point, approximately every 350 μm. The spread of results was relatively large, mainly due to irregularly occurring V_2_O_5_ crystallites, however the general relationship was clearly seen. The Bi:V ratio was close to one and more or less constant for films deposited at low pressures suggesting presence of BiVO_4_ phase. The Bi:V ratio lower than one confirmed the presence of the V_2_O_5_ phase. Higher pressure in the deposition chamber results in a compositional gradient across the layer and the Bi concentration increased (in comparison with V concentration) from the edge that was the closest to the target during deposition. EDX analysis confirmed the presence of Bi excess and formation of Bi_4_V_2_O_11_ for the films deposited under 2 mbar. The Bi:V ratio was not equal to 2, because two phases are present (BiVO_4_ and Bi_4_V_2_O_11_). The results obtained support the hypothesis that Bi and V atoms and ions in plasma plume can be separated due to the interactions or reaction with O_2_ gas, what affects the spatial distribution of elements on the deposited films.

**Figure 5:**
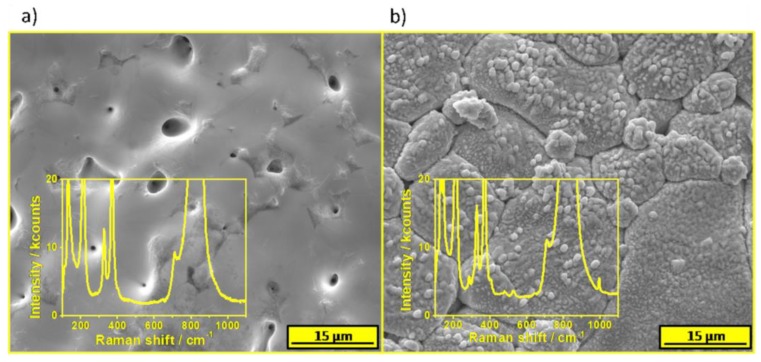
The SEM images and Raman spectra of a BiVO_4_ target after pulsed laser deposition. (**a**) The area exposed to the laser pulses and (**b**) the area not subjected to the ablation.

The morphology and composition of the spent target after deposition was investigated. The SEM micrograph of a target’s area exposed to laser pulses is presented in Figure 5a. Generally, the area is rather flat and BiVO_4_ particles cannot be distinguished, however some pinholes were formed. Raman spectrum of the area is characteristic of BiVO_4_ and V and Bi rich phases were not detected, see the inset of Figure 5a. It suggests that the ablation causes a formation of a plasma with a rather stoichiometric (Bi:V) composition. The pressure did not affect the ablation thresholds of V and Bi independently. The area of a target that was uncovered during deposition but not exposed to the laser pulses was also investigated. The SEM image is presented in Figure 5b. The surface was covered by a new phase, which was not observed before the PLD procedure (compared with Figure 1c). According to the Raman spectroscopy measurements, small particles contained the V_2_O_5_ phase as is shown in the inset of Figure 5b, however the main signal comes from the underlying BiVO_4_. Thus, vanadium was significantly affected by the oxygen gas in the chamber and it led to redeposition only of V_2_O_5_ on the target. Bi-rich phases were not detected there. Thus, it is very likely that the Bi/V separation occurs after ablation, during interactions with oxygen molecules with ions characterized by different masses.

**Figure 6:**
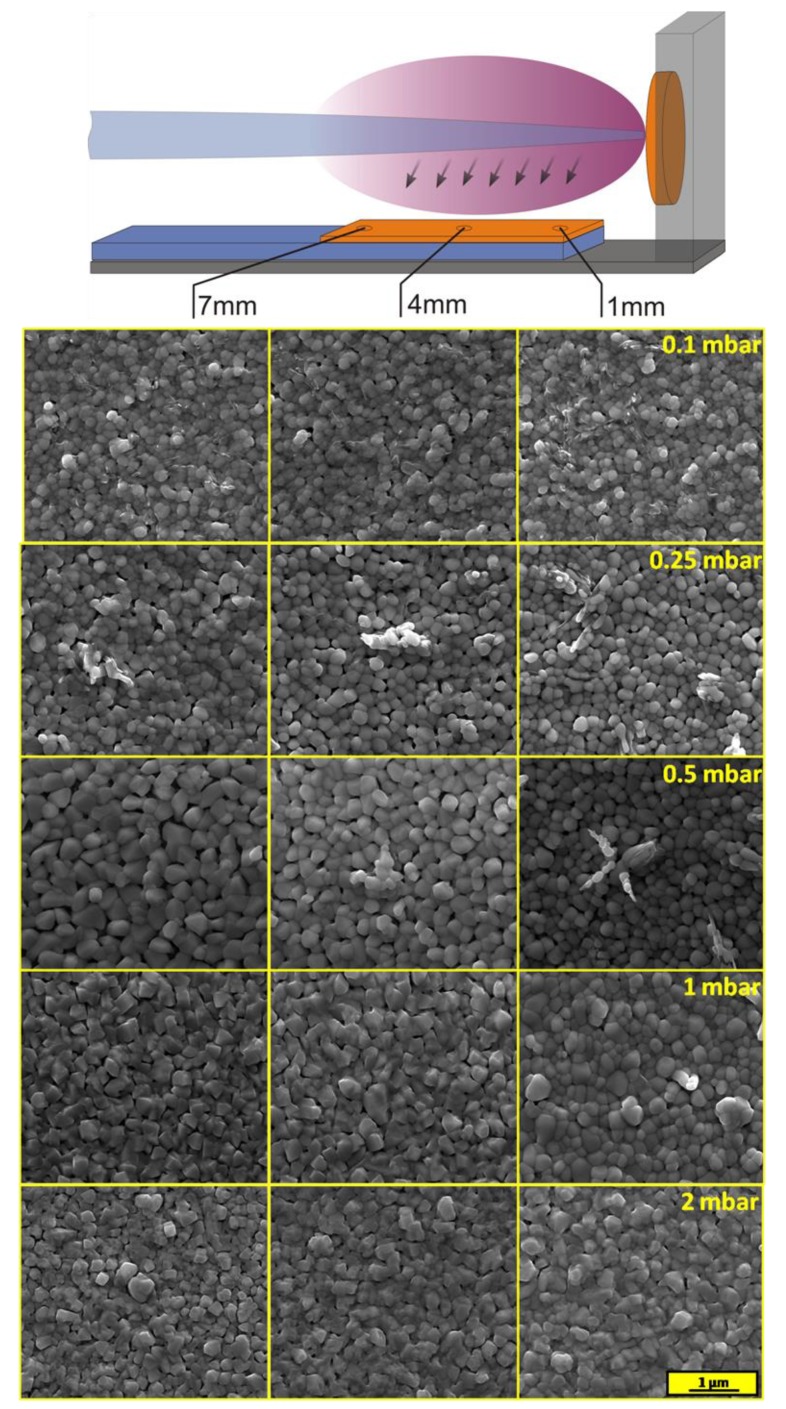
SEM images taken at different spots of the samples deposited under 0.1, 0.25, 0.5, 1, and 2 mbar.

The morphology of layers was investigated using scanning electron microscopy. SEM images were recorded at three different spots for each sample, as is shown in Figure 6. The sample deposited at the lowest pressure of O_2_ was quite homogenous, which was consistent with the Raman spectroscopy measurements. Films were built by round-shaped grains with different diameters from about 100 to 200 nm. Inclusion of another phase, probably V_2_O_5_, was also visible. The higher pressure during deposition affected the amount of the V_2_O_5_ phase on the film surface. Layers deposited under 0.5 mbar were built by clearly bigger particles of BiVO_4_. An additional phase of V_2_O_5_ on the top of BiVO_4_ was observed in a form of protruding longitudinal crystallites (confirmed by EDX analysis, see Figure S4), only near the edge of the sample that was the closest to the target. The cross-sectional micrograph of area rich in specific structures of the sample deposited under 0.5 mbar is presented in Figure S5. Although the V_2_O_5_ phase was also present on the surface of the samples deposited under 0.1 and 0.25 mbar (which was confirmed by Raman spectroscopy), the XRD technique enabled detection of it only in the case of the sample deposited under 0.5 mbar on which crystallites were formed. The effect of Bi to the V ratio on the morphology was visible on SEM images of the samples deposited under 1 and 2 mbar. The higher concentration of bismuth made the crystallite edges sharper, while a lower Bi concentration area was characterized by rather round-shape grains. It should be noticed that the separated phase of Bi_4_V_2_O_11_ could not be distinguished on the SEM micrographs. The SEM image of the larger area of the sample deposited under 2 mbar was added to the Supplementary Information in order to prove that there are no separated crystallites of Bi_4_V_2_O_11_, see Figure S6. It is very likely that the bulk Bi_4_V_2_O_11_@BiVO_4_ heterojunction was formed, as opposed to BiVO_4_/V_2_O_5_. It has been already shown previously that BiVO_4_ and Bi_4_V_2_O_11_ tend to form structures in which the components are indistinguishable (by SEM technique) due to the formation of nanosized interfacial contact [31]. Additionally, AFM measurements were performed In order to characterize the topography of the deposited films. The results are presented in Figure S7. The root mean square roughness of the sample deposited under 0.1 mbar was the lowest (32 nm), while the rest of the samples were characterized by rms equal to 38 nm.

**Figure 7:**
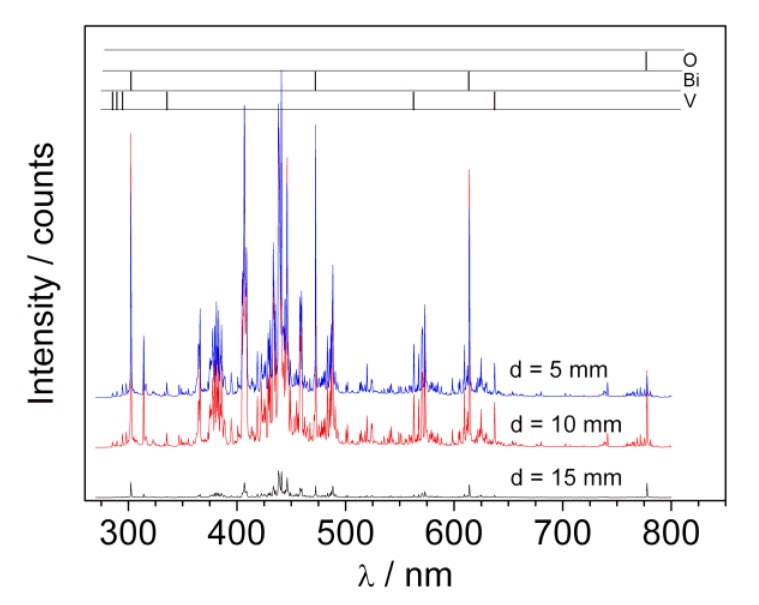
LIB spectra recorded during pulsed laser deposition process at different distances from target and oxygen pressure 2 mbar.

The plasma plume formed during deposition was also characterized using LIBS (laser-induced breakdown spectroscopy). The investigated radiation spectra contained clear atomic lines corresponding to oxygen, bismuth, and vanadium as well as continuous molecular spectra, which identification would require higher spectral resolution. Most probably these lines may correspond to oxygen and ozone molecules as well as bismuth and vanadium oxides or dimmers. The LIBS spectra recorded at different distances from the target during BiVO_4_ deposition (p = 2 mbar) are presented in Figure 7. The analyzed lines are marked in the upper part of the picture.

In the case of the signal coming from oxygen, there is a high intensity triplet O(777)—777.194, 777.417, and 777.539 nm (here seen as one line around 777 nm). The radiation results from optical transition *3s^5^S^0^_2_*→*3p^5^P_1 2 3_*. The energy of the higher transition level is around 10.74 eV and was not detected here. The bismuth lines observed in the spectra correspond to the transition from high energy levels *7s^2,4^P*, *8s^2^P*, and *6d^2^D* (with energies in the range of 4–6 eV) to metastable *6p^32^D^0^_3/2, 5/2_* and *6p^3 2^P^0^_1/2, 3/2_*. Lines corresponding to optical resonance transition (to ground electronic state *6p^3 4^S^0^_3/2_*) were not observed, which can be explained by full absorption in plasma volume. A radiation from vanadium atoms contains a large amount of spectral lines, which constitute the main part of the observed spectra. Energies of excited levels (*3d^3^4s4p ^4^D^0^*, *3d^4^4p ^4^P*, and *3d^4^4p ^4^F*) for observed lines were of the order 4 ÷ 5.3 eV. The observed transitions end in the ground electronic state (*3d^3^4s^2 4^F*) or metastable levels (*3d^4^4s ^6^D* and *3d^3^4s^2 4^P*). It should be underlined that in contrast to the bismuth spectra, in the case of vanadium the electrons fell to metastable as well as ground states. This means that the resonance lines of vanadium were reabsorbed in the plasma less than those of bismuth. It may point to the fact that there were significantly fewer atoms of vanadium than bismuth in the investigated plasma. Since Bi and V ablate from the target in a stoichiometric way, the results obtained indicate that propagation of vanadium in the deposition chamber filled with oxygen was therefore difficult.

**Figure 8:**
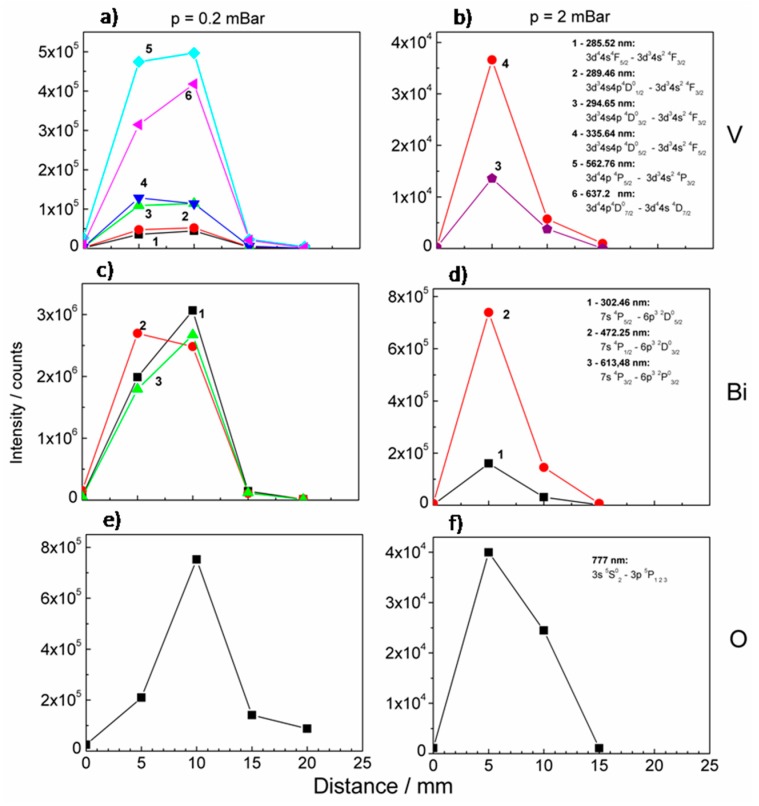
Line intensities as a function of distance from target for transitions in vanadium (**a**,**b**) 1—*3d^4^4s ^4^F_5/2_–3d^3^4s^2 4^F_3/2_*, 2—*3d^3^4s4p ^4^D^0^_1/2_–3d^3^4s^2 4^F_3/2_*, 3—*3d^3^4s4p ^4^D^0^_3/2_–3d^3^4s^2 4^F_3/2_*, 4—*3d^3^4s4p ^4^D^0^_5/2_–3d^3^4s^2 4^F_5/2_*, 5—*3d^4^4p ^4^P_5/2_–3d^3^4s^2 4^P_3/2_*, and 6—*3d^4^4p ^4^D^0^_7/2_–3d^4^4s ^4^D_7/2_*, for atomic bismuth (**c**,**d**) for transitions 1—*7s ^4^P_5/2_–6p^3 2^D^0^_5/2_*, 2—*7s ^4^P_1/2_–6p^3 2^D^0^_3/2_*,and 3—*7s ^4^P_3/2_–6p^3 2^P^0^_3/2_*, and for atomic oxygen (**e**,**f**) for triplet *3s^5^S^0^_2_*→*3p^5^P_1 2 3_*.

The intensities of the selected lines were plotted versus the distance from the BiVO_4_ target and the resulting graphs are presented in Figure 8. Measurements were performed under 0.2 and 2 mbar oxygen pressure. The dependence of intensities on a distance for low oxygen pressure was very similar for all observed vanadium lines, i.e., the intensities grew for a distance L < 10 mm and suddenly dropped later (Figure 8a). Similar dependences were observed in the case of bismuth (Figure 8c). As oxygen pressure grows the dependence changes. The maximum of intensities was shifted to lower L values, they grew for L < 5 mm and fell rapidly later, see Figure 8b,d. The dependences differed significantly in the case of oxygen both in the case of lower and higher oxygen pressure, see Figure 8e,f. Oxygen lines may originate from the oxygen ablated from the target. However, it is very likely that signals at about 777 nm originate from oxygen (ambient gas) species excited during interaction with plasma components.

During the laser ablation in the presence of ambient gas, the ablated species were decelerated and attenuated due to collision with gas molecules. These processes resulted in plasma confinement and generation of shock waves at the plasma propagation front, where chemical reactions of ablated materials with oxygen molecules were intensified. Higher ambient gas pressure results in the compression of plasma and shifting of the plasma front into the proximity of target. It was observed in the experiment as a change of spectral lines intensities versus the distance from a target measured for two different pressure values (Figure 8). For a higher pressure the maximum intensity of excited oxygen shifted towards the target, which means that the zone of intensive collision excitation also shift. The decrease of intensity beyond the maximum indicates a collision recombination. The attenuation of the emission with an increase of distance from the target may result from non-radiative processes (including quenching and energy transfer processes) and adiabatic expansion of the plasma. However the kinetic energy of all species was still sufficient to reach the substrate and form thin film. In our experiment, we observed the presence of the V_2_O_5_ phase, in a form of irregularly occurring crystallites, that for higher oxygen pressure was shifted towards the target. This was in an agreement with the spectroscopic observation of plasma properties. It should be also noted that the increase of ambient gas pressure resulted in the decrease of plasma plume species velocity, which can influence the morphology of the deposited film. For a higher pressure and distances from the target, where relaxation processes in plasma could be observed, the thin film was more homogenous, which was confirmed by SEM images (see Figure S6). The excess of vanadium in one part of the sample caused its deficiency in the area of the covered substrate, which was farther from the target. The effect of this phenomenon was a higher Bi:V ratio and possibility of Bi_4_V_2_O_11_ crystallization and formation of the BiVO_4_/Bi_4_V_2_O_11_ system.

**Figure 9:**
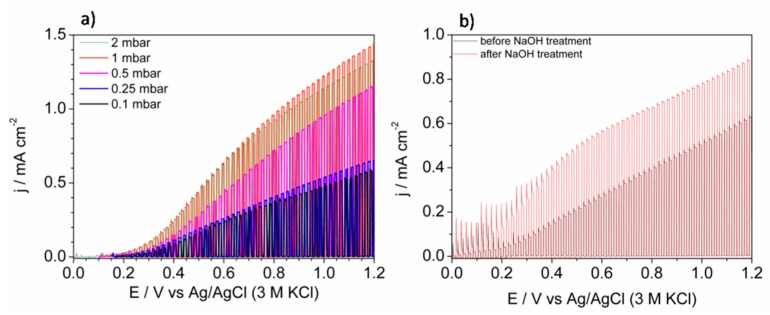
(**a**) The comparison of LSV curves recorded in 0.2 M K_2_SO_4_ under intermittent AM 1.5 illumination for FTO/BiVO_4_ photoanodes deposited under various oxygen pressures. (**b**) The comparison of LSV curves recorded for FTO/BiVO_4_ photoanodes (0.1 mbar) before and after NaOH treatment. Scan rate was 20 mV s^−1^.

The most promising application of thin layers of BiVO_4_ is photoelectrochemical water splitting. Thus, the obtained films were tested as photoanodes for water photooxidation under simulated solar light exposure. As it is shown in Figure 9a, the shape of linear sweep voltammograms was similar for each sample and the presence of heterojunctions (BiVO_4_/V_2_O_5_ and Bi_4_V_2_O_11_@BiVO_4_) did not clearly affect it. The curves were characteristic of n-type semiconductor electrodes under illumination [32]. The lowest photoactivity was observed for samples deposited under 0.1 mbar. Generally, the formation of BiVO_4_/V_2_O_5_ heterojunction can enhance the efficiency of photocurrent generation for both, layered and bulk types of junctions [10]. However, the geometry of the system significantly affects its photoactivity [33] due to the relative positions of conduction bands [34]. The photoexcited electrons can be trapped due to the potential barrier if the V_2_O_5_ is mainly on the top of the electrode, as it is in the present work. Thus, FTO/BiVO_4_ photoanode deposited under 0.1 mbar was immersed in 1 M NaOH in order to facilitate selective dissolution of V_2_O_5_. This is a procedure commonly used during some methods of BiVO_4_ synthesis that uses the excess of the V-containing precursor [35]. The comparison of the LSV (linear sweep voltammetry) curves recorded before and after soaking in NaOH solution is presented in Figure 9b. Since the enhancement of photocurrent was achieved, the presented results confirmed the negative influence of V_2_O_5_ on the surface on BiVO_4_. If BiVO_4_ and V_2_O_5_ did not form a heterojunction, an increase in photocurrent in comparison with BiVO_4_ would be expected due to the narrower energy band gap of vanadium pentaoxide. In the case of the sample deposited under 0.5 mbar, most of the sample surface consists of bare BiVO_4_. The enhancement of the photocurrent, in comparison with samples deposited under lower pressures, results from the lack of adverse BiVO_4_/V_2_O_5_ junction presence on a larger area of the film. The highest photocurrent of water oxidation was observed for the sample deposited under 1 mbar, however the system was very complex due to the presence of three phases: BiVO_4_, V_2_O_5_, and Bi_4_V_2_O_11_. A very similar result was achieved for the sample that consisted of the Bi_4_V_2_O_11_@BiVO_4_ heterojunction (deposited under 2 mbar). Notably, pristine FTO/BiVO_4_ photoanodes (prepared under 0.1 mbar with removed V_2_O_5_) generated a lower photocurrent than photoanodes consisting of the BiVO_4_@Bi_4_V_2_O_11_ heterojunction. It has been already reported that such a system positively affects the photoelectroactivity in comparison to bare BiVO_4_ [31,36]. The geometry of the system should be taken into account, as it was discussed for the BiVO_4_/V_2_O_5_ photoanode. However, the crystallites of Bi_4_V_2_O_11_ cannot be simply distinguished on the SEM images, thus it is very likely that bulk heterojunction was obtained. Since the pulsed laser deposited films of Bi_4_V_2_O_11_ acts as a rather poor photoanode i.a. due to the low absorption coefficient [37], in the case of the formation of BiVO_4_ and Bi_4_V_2_O_11_ separated phases, the enhancement would not be expected. Thus, the formation of heterojunction is the main reason of photocurrent improvement. The formation of heterojunction leads to the inhibition of bulk electron/hole recombination [38]. Generally, the presence of type-II heterojunction enhances bulk e^−^/h^+^ separation efficiency due to the internal electric field generated on the phases interface, e.g., BiVO_4_/Bi_4_V_2_O_11_ [36]. The enhancement of photoactivity in the case of the Bi_4_V_2_O_11_-containing photoanode may be also related to improved charge transfer resistance through the photoactive film in comparison with pristine BiVO_4_ as it was reported previously [22]. It is not expected that improvement is related to better light absorption because the energy band gaps of BiVO_4_ and Bi_4_V_2_O_11_ are comparable [39]. Since the morphology, thickness, and roughness of the samples were comparable, we claimed that differences in the photocurrent of water oxidation were related mainly to the composition of the samples. The recorded photocurrent seems to be lower in comparison with other pulsed laser deposited BiVO_4_-based photoanodes [22,40,41]. However it should be noticed that in the case of the presented results the photocurrent was related to water photooxidation and oxygen evolution, not oxidation of the consumable hole scavenger. On the other hand, results were comparable with the photocurrent of water oxidation that was achieved for unmodified BiVO_4_ films with underlying hole-blocking layer sputtered using the PLD method [42]. 

## 4. Summary

In summary, we successfully performed the deposition of photoactive films on FTO substrates from the BiVO_4_ target using the pulsed laser deposition technique. The influence of oxygen pressure in a deposition chamber on composition, morphology, and photoelectrochemical properties of layers was studied. It was shown that BiVO_4_/V_2_O_5_ heterojunction could be formed in the case of lower O_2_ pressures (0.1, 0.25 0.5, and 1 mbar), while Bi_4_V_2_O_11_@BiVO_4_ bulk heterojunction was formed in the case of higher oxygen pressures (1 and 2 mbar). Comparing the results of XRD, Raman spectroscopy, EDX, and SEM, it could be concluded that in the case of low pressures, the distribution of the additional V_2_O_5_ phase was quite homogeneous, and for moderate pressures separate V_2_O_5_ structures were formed in some areas of the investigated films. In the case of the films deposited at high oxygen pressures, the morphology of the area that contained BiVO_4_@Bi_4_V_2_O_11_ differed from bare BiVO_4_, however, the new, separated phase could not be distinguish on the SEM images, suggesting the formation of a well distributed BiVO_4_@Bi_4_V_2_O_11_ heterojunction. The formation of new phases (V_2_O_5_ and Bi_4_V_2_O_11_) was strictly related to the pressure and the location of the investigated area on the sample. According to our results, it could be concluded that the stoichiometry and composition could be tuned by changing the oxygen pressure in the deposition chamber and the distance of the substrate from the target during sputtering. The characterization of the target performed after exposure to laser pulses showed that Bi and V ablate from the target in a stoichiometric way, thus the separation of Bi and V atoms occurred in a plasma plume due to interactions with oxygen molecules. As a result, there were areas on the deposited samples that were enriched in V (BiVO_4_/V_2_O_5_) and Bi (Bi_4_V_2_O_11_@BiVO_4_), depending on the distance from the target during the deposition. According to the LIBS measurements, there were transition lines characteristic for vanadium, bismuth, and oxygen. Unlike bismuth transitions, vanadium-specific transition lines end in a ground-state, suggesting that there were fewer V atoms in plasma plume at measurements spots. The photoanodes prepared under different oxygen pressures exhibited different photoelectrochemical properties. In the case of FTO/BiVO_4_/V_2_O_5_ films deposited under low pressure, photoactivity measured as a photocurrent of water oxidation was worse in comparison to FTO/Bi_4_V_2_O_11_@BiVO_4_ photoanodes obtained under higher O_2_ pressure. The presence of the Bi_4_V_2_O_11_@BiVO_4_ heterojunction positively affected the photocurrent in comparison with pristine BiVO_4_ photoanodes as well.

## 5. Conclusions

The formation of V_2_O_5_ and Bi_4_V_2_O_11_ phases during BiVO_4_ deposition using the pulsed laser deposition technique was strictly related to the oxygen pressure and the location of the investigated area on the sample. According to our results, it could be concluded that the stoichiometry and composition could be tuned by changing the oxygen pressure in the deposition chamber and the distance of the substrate from the target during sputtering. The problem with non-stoichiometric deposition of multielemental materials could be related to the different scattering and interaction of ablated ions with gas molecules present in the chamber. Among the tested electrodes, the highest photoactivity was achieved for the samples that consisted of the BiVO_4_/Bi_4_V_2_O_11_ bulk heterojunction.

## Supplementary Materials

The following are available online at https://www.mdpi.com/1996-1944/13/6/1360/s1, Figure S1: The self-made system for pulsed laser deposition. Figure S2: The exemplary cross-sectional SEM micrograph of the sample deposited under 0.1 mbar. Figure S3: The small-angle XRD patterns of the samples deposited under 0.1, 0.5, and 2 mbar. Figure S4: The EDX analysis of the V_2_O_5_ crystals formed on the BiVO_4_ film deposited under 0.5 mbar. Figure S5: The cross-sectional SEM micrograph of the area rich in longitudinal crystallites. Figure S6: The SEM image of the sample deposited under 2 mbar. Figure S7: AFM topography of films deposited under 0.1, 0.25, 0.5, 1, and 2 mbar. Table S1: The variation of the film thickness depending on the oxygen pressure and the distance from the target.
