# Supplementary material for: Pulsed Laser Deposition of Bismuth Vanadate Thin Films—The Effect of Oxygen Pressure on the Morphology, Composition, and Photoelectrochemical Performance
Source: Materials (Basel). 2020 Mar 17;13(6):1360. doi: 10.3390/ma13061360 (PMC7143622; doi:10.3390/ma13061360)
Supplement: Supplementary file 1 [file materials-13-01360-s001.pdf]

Supplementary Materials

Pulsed Laser Deposition of Bismuth Vanadate Thin Films—The Effect of Oxygen Pressure on the Morphology, Composition, and Photoelectrochemical Performance

Konrad Trzciński, Mariusz Szkoda, Maria Gazda, Jakub Karczewski, Adam Cenian, Galina M. Grigorian and Mirosław Sawczak

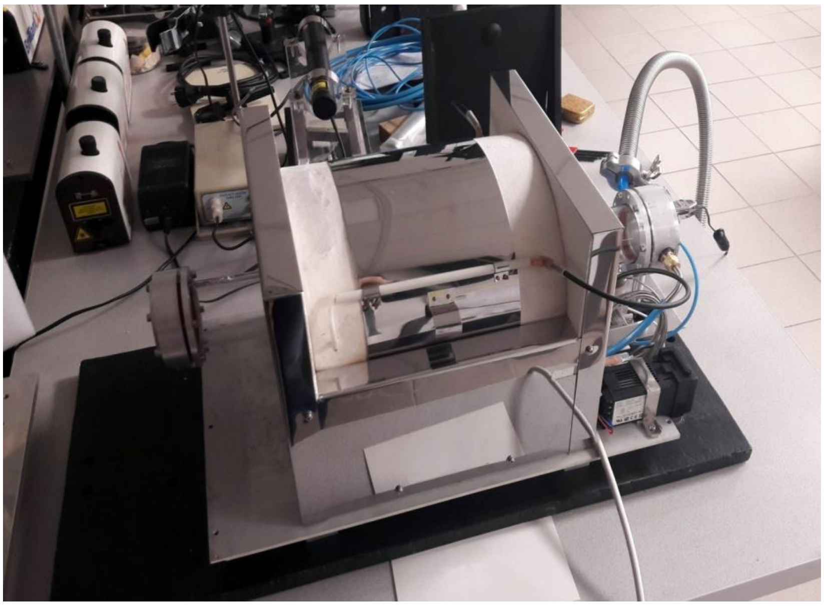

Figure S1. The self-made system for pulsed laser deposition.

Table S1. The variation of the film thickness depending on the oxygen pressure and the distance from the target,.

	oxygen pressure				
distance from the target	0.1 mbar	0.25 mbar	0.5 mbar	1 mbar	2 mbar
5 mm	130 nm	150 nm	150 nm	130 nm	130 nm
7 mm	160 nm	180 nm	170 nm	140 nm	160 nm
9 mm	160 nm	180 nm	150 nm	160 nm	160 nm
11 mm	160 nm	200 nm	160 nm	160 nm	160 nm
13 mm	140 nm	180 nm	150 nm	140 nm	160 nm

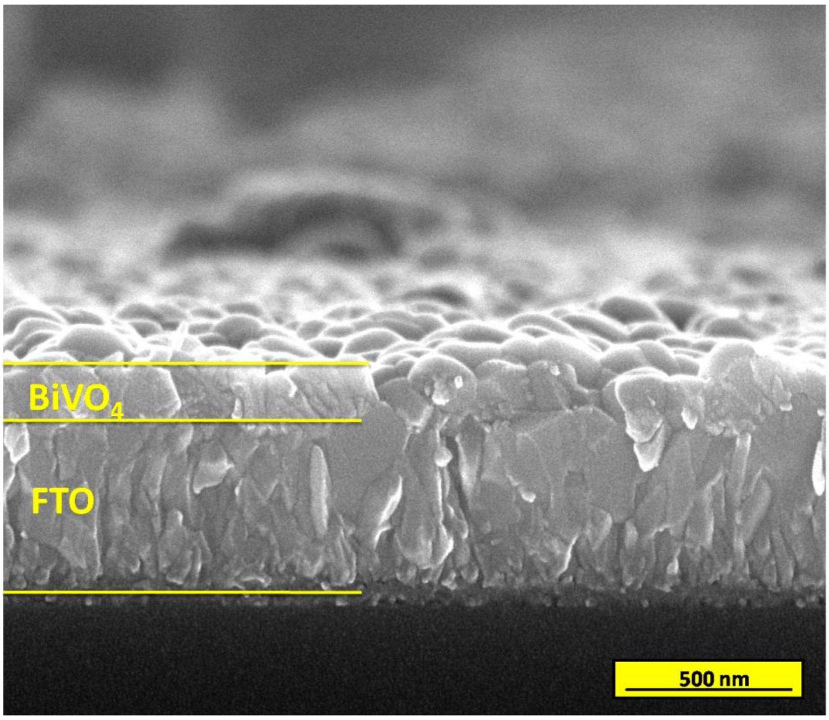

Figure S2. The exemplary cross-sectional SEM micrograph of the sample deposited under 0.1 mbar.

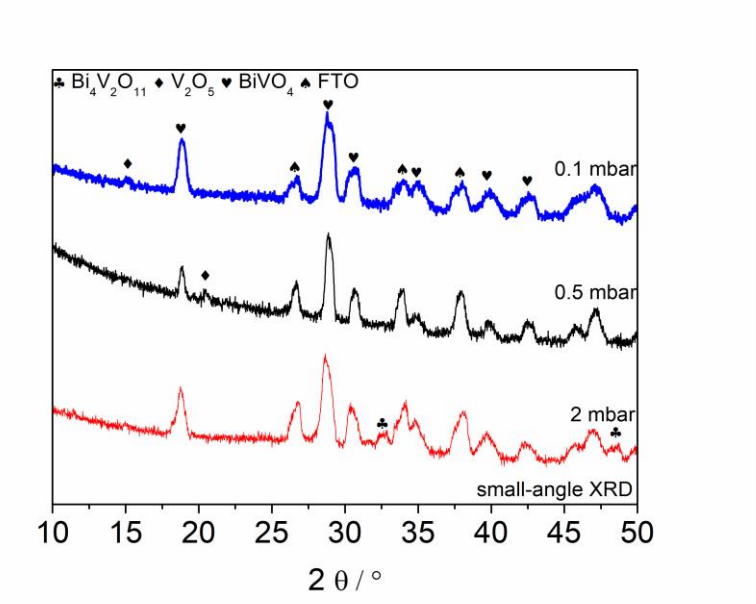

Figure S3. The exemplary small-angle XRD patterns of the samples deposited under 0.1, 0.5, and 2 mbar.

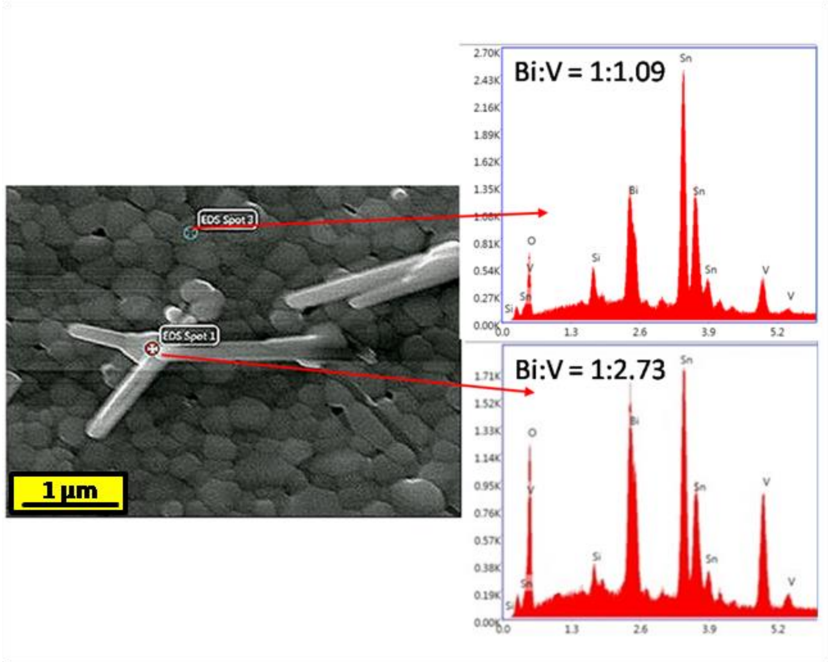

Figure S4. The EDX analysis of the V_2O_5 crystals formed on the $BiVO_4$ film deposited under 0.5 mbar.

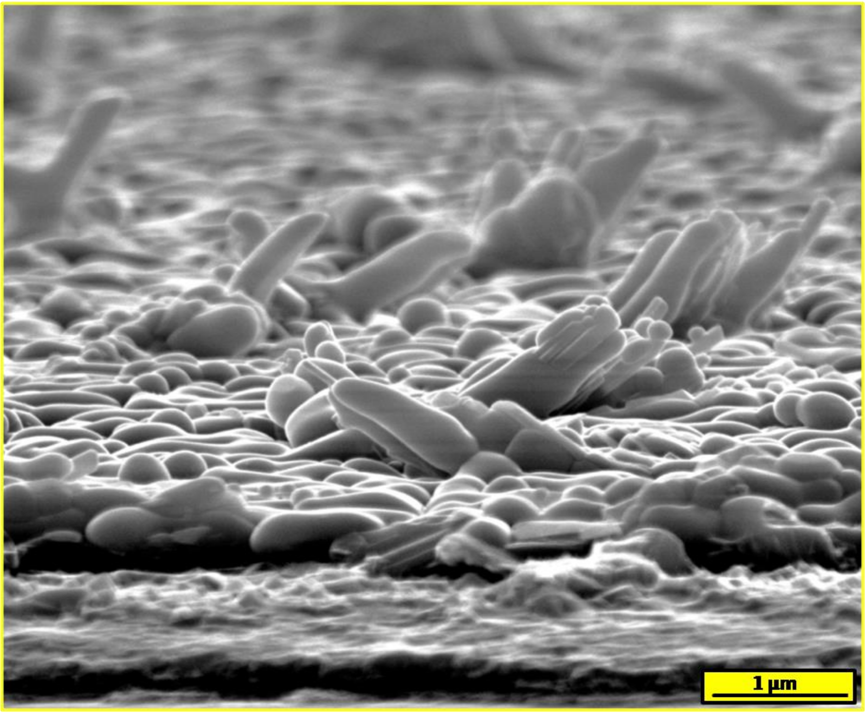

Figure S5. The cross-sectional SEM micrograph of the area rich in longitudinal crystallites.

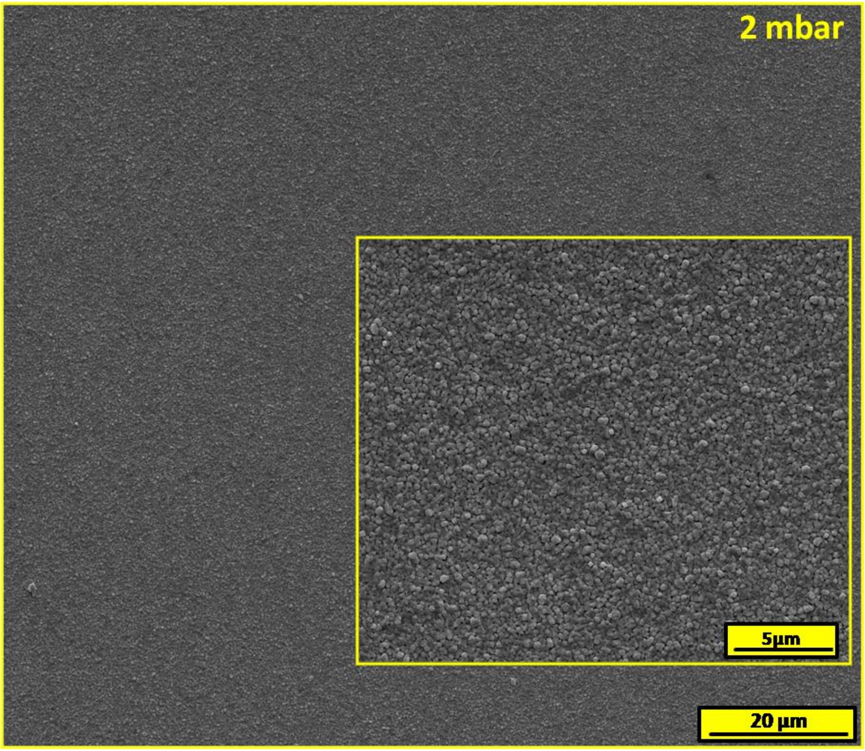

Figure S6. The SEM image of the sample deposited under 2 mbar.

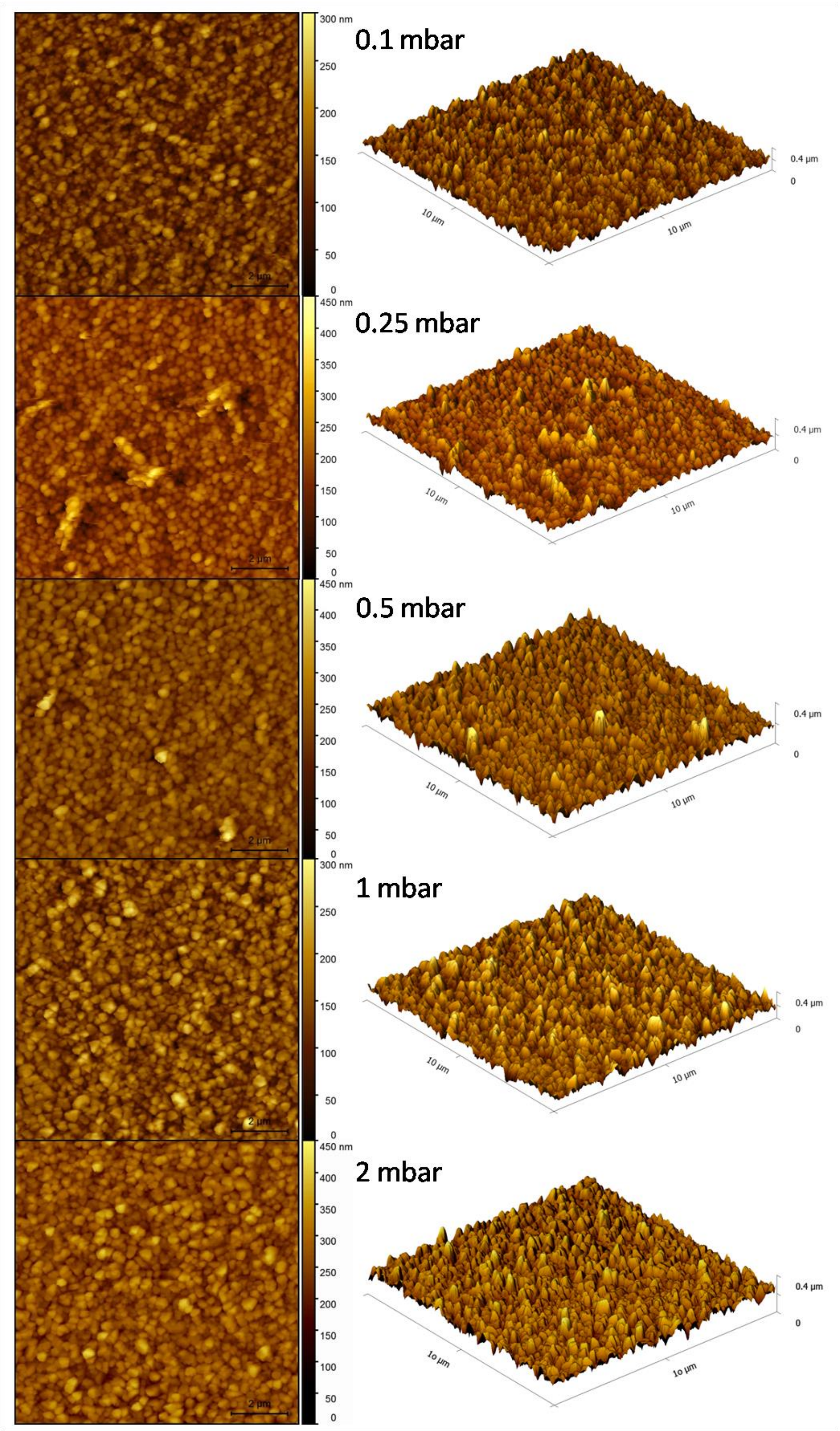

Figure S7. AFM topography of films deposited under 0.1, 0.25, 0.5, 1, and 2 mbar.
